# Epigenetic regulation of individual components of combined hepatocellular-cholangiocarcinoma

**DOI:** 10.1371/journal.pone.0324145

**Published:** 2025-05-27

**Authors:** Kyle M. Schachtschneider, Luke N. Redlon, Ryan Peter Lokken, Yu-Hui Huang, Grace Guzman, Lawrence B. Schook, Ron C. Gaba

**Affiliations:** 1 Department of Radiology, University of Illinois at Chicago, Chicago, Illinois, United States of America; 2 Department of Biochemistry and Molecular Genetics, University of Illinois at Chicago, Chicago, Illinois, United States of America; 3 National Center for Supercomputing Applications, University of Illinois at Urbana-Champaign, Urbana, Illinois, United States of America; 4 College of Medicine, University of Illinois at Chicago, Chicago, Illinois, United States of America; 5 Department of Radiology and Biomedical Imaging, University of California, San Francisco, California, United States of America; 6 Department of Radiology, University of Minnesota, Minneapolis, Minnesota, United States of America; 7 Department of Pathology, University of Illinois at Chicago, Chicago, Illinois, United States of America; 8 Department of Animal Sciences, University of Illinois at Urbana-Champaign, Urbana, Illinois, United States of America; Regeneron Pharmaceuticals Inc, UNITED STATES OF AMERICA

## Abstract

Combined hepatocellular carcinoma-cholangiocarcinoma (HCC-CCA) is a rare liver tumor comprising histologic features of both HCC and CCA. Due to its heterogeneous nature, treatment of combined HCC-CCA is a significant clinical challenge and prognosis remains poor. Therefore, further understanding of the tumor biology underlying the individual subtypes of this mixed tumor is required to improve treatment stratification and optimize treatment strategies. This study sought to identify altered epigenetic regulation and gene expression patterns in the individual components of combined HCC-CCA. Formalin fixed paraffin embedded (FFPE) tumor specimens from 9 patients diagnosed with combined HCC-CCA were utilized in this study. Hematoxylin and eosin (H&E) staining was performed for each sample, and regions representative of the individual HCC and CCA components were delineated. Adjacent unstained slides were cut and dissected to separate HCC and CCA components. DNA and RNA extraction was performed for each sample for DNA methylation (n = 7 HCC and 7 CCA) and gene expression (n = 7 HCC and 8 CCA) profiling via reduced representation bisulfite sequencing (RRBS) and RNA-seq, respectively. Samples did not cluster by tumor type when comparing genome-wide DNA methylation or gene expression patterns. Of the 5 patients with DNA methylation data available for both subtypes, 4 clustered by patient as opposed to cancer subtype, suggesting similar epigenetic regulatory patterns arising from development in the same microenvironment and genetic background. Differential analysis resulted in the identification of 57 differentially expressed genes (DEGs) and 808 differentially methylated regions (DMRs) between the HCC and CCA subtypes. Genes associated with DMRs were associated with Wnt signaling, voltage-gated channels, metal binding, and cellular regulation. Finally, increased expression of several genes previously implicated in tumor aggressiveness, prognosis, and treatment responses were identified. These results highlight the potential importance of accounting for underlying HCC and CCA tumor biology when determining the optimal course of treatment for this deadly disease.

## Introduction

Combined hepatocellular carcinoma-cholangiocarcinoma (HCC-CCA) is a rare liver tumor comprising histologic features of both HCC and CCA [1]. This disease is thought to arise from hepatic stem cells capable of both hepatic and ductal differentiation [2,3] or de-differentiation or trans-differentiation of primary HCC or CCA [4]. Mutational drivers of HCC-CCA tumorigenesis include *TP53*, *TERT*, and various receptor tyrosine kinase, cell cycle, and Wnt pathway genes [5]. While the reported incidence of combined HCC-CCA ranges from 0.4–14.2%, the true incidence may be underestimated due to the lack of biopsy or surgical resection for definitive histological analysis [6], deficiency of consistently expressed serologic tumor markers [7], non-specific radiologic imaging findings [7–9], and potential misdiagnosis as isolated HCC or CCA [10,11]. Due to its heterogeneous nature, management of combined HCC-CCA is a significant clinical challenge, and prognosis remains poor [12–14]. Despite treatment options spanning systemic chemotherapy, locoregional therapy, hepatectomy, and liver transplantation, overall survival rates remain dismal, ranging from 8–40% for palliative to curative therapies, respectively [15,16].

Application of functional genomics—or alterations in epigenetic and gene expression patterns [17]—offers the potential to further understand the tumor biology underlying the individual subtypes of HCC-CCA to facilitate treatment stratification and optimize therapeutic strategies. To this end, previous studies investigating gene expression in combined HCC-CCA suggest a genetic profile more similar to CCA than HCC, potentially explaining the similar prognosis [18]. However, given the rarity of HCC-CCA, additional data are necessary to support further insights into this disease. As such, this study sought to identify the epigenetic regulation underlying gene expression patterns in the individual components of combined HCC-CCA.

## Materials and methods

### Patient population

**Fig 1:**
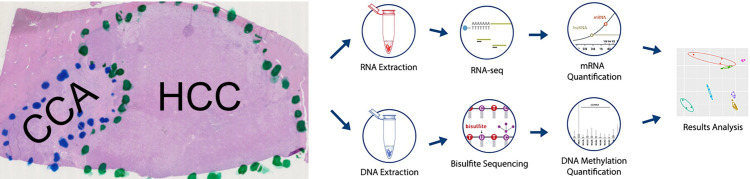
Overall study workflow. Histology slides were dissected to separate HCC and CCA components, genetic material was then extracted, sequenced, and quantified, and functional analysis was then performed.

This single-center retrospective study, including chart review and waiver of written informed consent, was approved by the University of Illinois at Chicago (UIC) Institutional Review Board (IRB) and was in compliance with the Health Insurance Portability and Accountability Act (HIPAA). The cohort included formalin fixed paraffin embedded (FFPE) biopsy (n = 3) and resected (n = 6) tumor specimens from 9 male patients (age range 49–69 years old) diagnosed with combined HCC-CCA. Overall study workflow is depicted in Fig 1.

### Histological evaluation and tissue processing

FFPE tumor blocks were obtained from the UIC Biorepository and provided to the UIC Research Histology and Tissue Imaging Core for sectioning and staining. Hematoxylin and eosin (H&E) staining was performed for each sample, and regions representative of the individual HCC and CCA components were delineated by a board-certified human pathologist with subspecialty training in Liver and Transplantation Pathology. Adjacent unstained slides were sterilely cut, mounted on glass slides, and stored at –80 °C until processing. Using the annotated H&E stained slides as a guide, unstained frozen slides were dissected to separate HCC and CCA components, which were placed in separate microcentrifuge tubes for deoxyribonucleic acid (DNA) and ribonucleic acid (RNA) extraction. HCC and CCA components were present on dissected slides for all but 3 patients, who only had either HCC (n = 1) or CCA (n = 2) components present.

### DNA and RNA extraction

DNA and RNA were simultaneously extracted from each sample using the AllPrep DNA/RNA FFPE Kit (Qiagen, Valencia, CA) following the manufacturer’s instructions. RNA and DNA was then provided to the Carver High-Throughput DNA Sequencing and Genotyping Unit (HTS lab, University of Illinois, Urbana, IL) for quantification on a Qubit (Life Technologies). RNA quantifications ranged from 133 ng to 13.75 μg, while DNA quantifications ranged from 13 ng to 5.04 μg. DNA extraction for the CCA component of one patient was unsuccessful, and therefore this sample was not used for DNA methylation analysis.

### Illumina sequencing

DNA methylation (n = 7 HCC and n = 7 CCA) and gene expression (n = 7 HCC and n = 8 CCA) patterns were profiled via reduced representation bisulfite sequencing (RRBS) and RNA-seq, respectively. RNA-seq libraries were prepared by the HTS lab using the FFPE mRNAseq library construction kit (NuGEN). Single end 150 bp reads were produced on an Illumina HiSeq4000. RRBS libraries were prepared by the HTS lab using the Ovation RRBS MethylSeq System Library Preparation Kit (NuGEN). Single end 100 bp reads were produced on an Illumina HiSeq4000. The data sets supporting the results of this article are available in the Sequence Read Archive under accession number PRJNA1236834.

Although numerous previous studies have demonstrated the ability to successfully perform sequence analyses on DNA and RNA isolated from FFPE tumor samples [19–22], nucleotide degradation is a known issue when working with FFPE samples [23]. Therefore, several quality assessments were performed to investigate the quality of the RNA-seq and RRBS datasets utilized in this study.

### RNA-seq data analysis

On average, 36 million reads were produced for each library (range 17 to 52.5 million). Raw reads were trimmed for adaptors, A-tails, quality, and length using trim_galore V0.4.4 as previously described [24,25]. Trimmed reads were aligned to the human reference genome (GRCh38, annotation 38.91) using STAR V2.5.3a [26] in 2-pass mode, setting the --sjdbOverhang option to 149 and the --limitSjdbInsertNjs option to 1500000. The Picard V2.10.1 CollectRnaSeqMetrics tool was used to assess 3’ bias [27], which was corrected when estimating expression levels using rsem-calculate-expression (RSEM V1.3.0 [28]) with the following parameters: --seed-length 5, --strandedness forward, and including the --estimate-rspd option. Finally, RSEM results files were imported into R V3.5.1 [29] using tximport [30] for differential gene expression analysis. DESeq2 [31] was used to identify differentially expressed genes (DEGs) between CCA and HCC samples, and genes with a q-value < 0.05 were considered differentially expressed.

### RRBS data analysis

On average, 51 million reads were produced for each library (range 23.1 to 75.6 million). Raw reads were trimmed for adaptors, quality, and length using trim_galore V0.4.4. Additional sequences added by the diversity adaptors were then removed using the trimRRBSdiversityAdaptCustomers.py custom python script (NuGEN). Following trimming, reads were aligned to the human reference genome (GRCh38) using BSseeker V2.1.2 [32] with the following parameters: -m 2, --aligner = bowtie2, --bt2-local, --bt2-N 1, and --bt2-L 20. Methylation calls were extracted using the bs_seeker2-call_methylation.py script. As mitochondrial genomes harbor little to no DNA methylation, reads aligning to the mitochondrial genome included in the human reference genome (GRCh38) were used to calculate the bisulfite conversion rate for each sample. Bisulfite conversion rates were calculated by dividing the number of unmethylated reads by the total number of reads aligning to a given cytosine on the mitochondrial genome, with conversion rates ranging from 98.58% to 99.01%. Single nucleotide polymorphisms (SNPs) were identified using BS-SNPer [33] with the following parameters: --minhetfreq 0.1, --minhomfreq 0.85, --minquali 15, --mincover 10, --maxcover 10000, --minread2 2, --errorate 0.02, and --mapvalue 20. SNPs were removed using custom python scripts. In order to reduce potential sequencing depth biases, downstream analyses were limited to CpG sites covered by a minimum of 10 reads (high confidence sites). High confidence CpG sites were then utilized for differential methylation analysis using methylKit [34] after removing sites with high read coverage (upper 99.9^th^ percentile) using the filterByCoverage function and normalizing coverage across samples using the normalizeCoverage function. Sites were grouped into 200 bp regions using the tileMethylCounts function for differential methylation analysis, and regions were considered differentially methylated regions (DMRs) with a minimum methylation difference of 25% and a q-value < 0.05. DMRs overlapping genes and regulatory regions were identifying using Ensembl annotation version 38.91.

### Functional annotation analysis

Functional annotations enriched for DEGs and genes associated with DMRs were determined using DAVID v6.8 using default categories [35,36]. The Benjamini-Hochberg method was used for multiple testing correction of P-values, with Gene Ontology (GO) terms and pathways with a q-value < 0.05 considered significantly enriched.

### Clinical data

Clinical data were extracted from the institutional electronic medical record. data were accessed for research purposes between June 30, 2016 and September 30, 2024. Some investigators had access to information that could identify individual participants during collection.

### Statistical analysis

R V3.5.1 [29] was utilized for statistical analyses. Correlation analysis was performed using the Spearman’s correlation coefficient, and ANOSIM was performed using Euclidean dissimilarities. Enrichments were tested for significance using a Chi-square test. Significance for all tests was based on a P-value < 0.05.

## Results

### Patient demographics

Features of the study population are presented in Table 1. All subjects were quadragenarian to sexagenarian men. Liver disease etiology and stage were variable, though most patients had normal functional status. Tumor features are presented in Table 2.

**Table 1:** Patient demographics and liver disease characteristics.

Measure	Value ^a^
Patients	7
Sex	
Male	7 (100%)
Age (years)	59 ± 9 (49-69)
Liver disease etiology	
Hepatitis C virus	3 (43%)
Non-alcoholic steatohepatitis	2 (29%)
Alcohol	1 (14%)
No liver disease	1 (14%)
ECOG performance status	
0	6 (86%)
4	1 (14%)
Child-Pugh score	8 ± 4 (6-14)
Class A	4 (57%)
Class B	1 (14%)
Class C	2 (29%)

Legend: This table displays features of the patient population.

^a^ Number (%) or mean ± standard deviation (range)

**Table 2:** Tumor features.

Measure	Value ^a^
Tumor focality	
Solitary	2 (29%)
Oligonodular	1 (14%)
Multinodular	4 (57%)
Tumor number	5 ± 3 (1-7)
Largest tumor size (cm)	5.3 ± 1.8 (3.2-8.0)
Tumor histological pattern	
Pseudoglandular	3 (43%)
Trabecular	2 (29%)
Solid	1 (14%)
Not reported	1 (14%)
Tumor cytology	
Usual	4 (57%)
Clear cell	2 (29%)
Not reported	1 (14%)
Edmondson-Steiner grade	
2	5 (71%)
3	2 (29%)
Alpha fetoprotein (ng/dL)	2,861 ± 7,445 (1.6-19,744)
BCLC stage	
Early stage (A)	1 (14%)
Intermediate stage (B)	2 (29%)
Advanced stage (C)	2 (29%)
Terminal stage (D)	2 (29%)
Milan criteria	
Within	1 (14%)
Outside	6 (86%)

Legend: This table displays characteristics of the included tumors.

^a^ Number (%) or mean ± standard deviation (range)

### Genome-wide transcriptional and DNA methylation profiles

**Fig 2:**
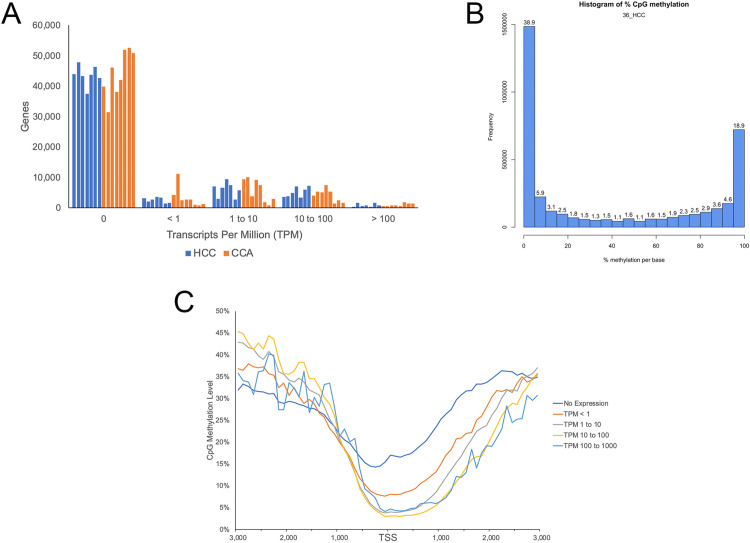
Global DNA methylation and gene expression profiles. (A) Gene expression levels for all annotated genes for each sample. (B) Representative histogram displaying the percent methylation of all covered CpG sites. (C) Average CpG methylation levels surrounding gene TSSs, demonstrating a negative correlation between CpG methylation and gene expression.

Alignment rates ranged from 62.69% to 93.96% for RNA-seq samples (S1 Table), and 66.84% to 79.59% for RRBS samples (S1 Table). Varying levels of 3’ bias were observed across RNA-seq samples, suggesting RNA degradation resulting from the use of FFPE samples [37]. This 3’ bias was corrected during quantification. In addition, a high proportion of annotated genes displayed no expression (transcripts per million, TPM = 0), ranging from 53.76% to 90.4% of annotated genes, compared to 8.09% to 27.2% of genes displaying an expression value ≥ 1 TPM (Fig 2A, S1 Table). A bimodal distribution of DNA methylation levels was observed for all samples (Fig 2B), typical of previously reported RRBS studies [24]. In addition, DNA methylation levels at transcription start sites (TSS) were negatively correlated with gene expression in all samples (Spearman’s rho = –0.133 to –0.546; P < 2.2 x 10^-16^; Fig 2C). Together, these results suggest high levels of RNA degradation stemming from the use of FFPE samples in this study. However, as the expected negative correlation between TSS methylation and gene expression was observed across samples, and no differences in quality were observed between HCC and CCA samples, comparative analyses were performed.

### Differential expression associated with individual HCC-CCA components

**Fig 3:**
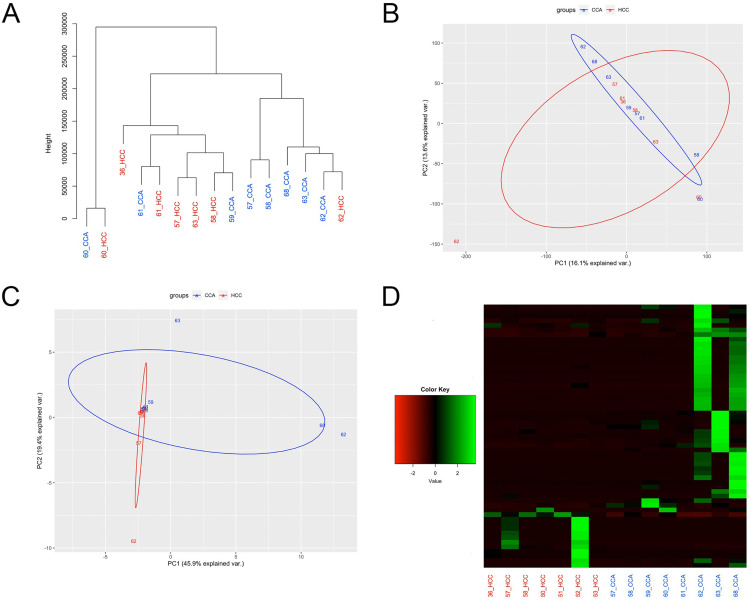
Differential expression between HCC and CCA components. (A) Cluster analysis and (B) PCA plot based on gene expression levels of all annotated genes. (C) PCA plot based on gene expression levels of the 57 DEGs. (D) Heatmap of the expression levels for the 57 DEGs for each sample, represented as z-scores.

Although cluster analysis demonstrated samples tended to cluster by tumor type based on genome-wide expression profiles, with 5 of 8 CCA and 4 of 7 HCC samples clustering together (Fig 3A), tumor types were not distinguishable based on PCA analysis (ANOSIM R = –0.046, P-value = 0.89; Fig 3B). Differential gene expression analysis resulted in the identification of 57 DEGs between the HCC and CCA components (S3 Table), with 46 upregulated and 11 downregulated in the CCA compared to HCC components. Samples did not cluster by tumor type when comparing the expression level of the 57 DEGs (ANOSIM R = 0.052 P-value = 0.131; Fig 3C), likely due to the high level of within group variability in expression (Fig 2D).

While functional analysis did not result in identification of any enriched terms or pathways, increased expression of genes associated with tumor progression, aggressiveness, and survival was observed in CCA compared to HCC components, including *LPAR3*. *NEFL* was upregulated in CCA compared to HCC components. Increased expression of *TUSC7* was also observed in the CCA compared to HCC samples. Increased expression of the transcriptional regulator *NANOG* was also observed in the CCA samples.

Increased expression of several genes associated with treatment responses was also observed in CCA compared to HCC components, including *TYRO3*. *BRWD1* expression was also increased in CCA compared to HCC samples. Finally, increased *TTBK2* expression was observed in CCA compared to HCC samples.

### Differential methylation associated with individual HCC-CCA components

**Fig 4:**
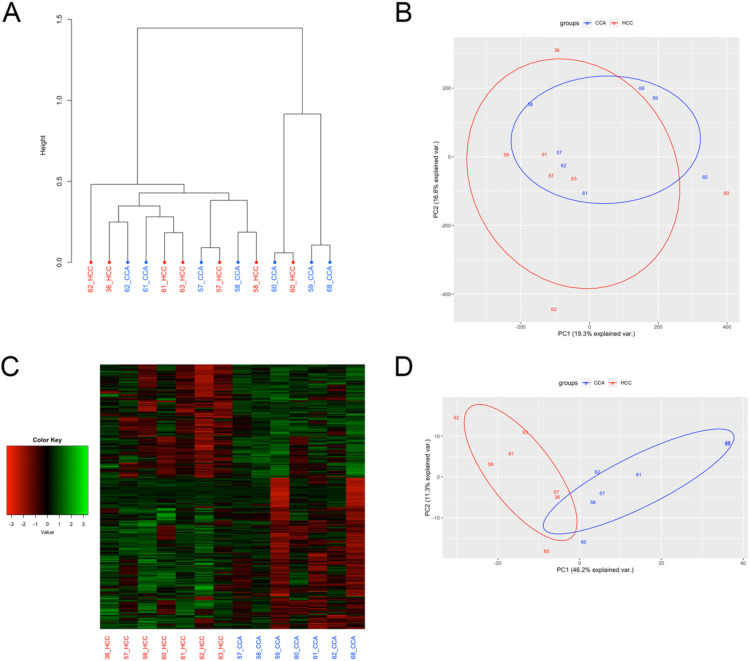
Differential methylation between HCC and CCA components. (A) Cluster analysis and (B) PCA plot based on DNA methylation levels of all tested regions. (C) Heatmap of the DNA methylation levels for the 808 DMRs for each sample, represented as z-scores. (D) PCA plot based on DNA methylation levels of the 808 DMRs.

Similar to the genome-wide gene expression profiles, samples did not cluster by tumor type when comparing genome-wide DNA methylation patterns (ANOSIM R = 0.04, P-value = 0.245; Figs 4A, B). Interestingly, of the 5 patients with DNA methylation data available for both subtypes, 4 clustered by patient as opposed to cancer subtype, suggesting similar epigenetic regulatory patterns arising from development in the same microenvironment and genetic background. Differential methylation analysis resulted in the identification of 808 DMRs, with 347 hyper- and 461 hypomethylated in the CCA compared to HCC samples (Fig 4C). Samples clustered by tumor type when comparing DNA methylation levels of the 808 DMRs (ANOSIM R = 0.43, P-value = 0.001; Fig 4D).

**Fig 5:**
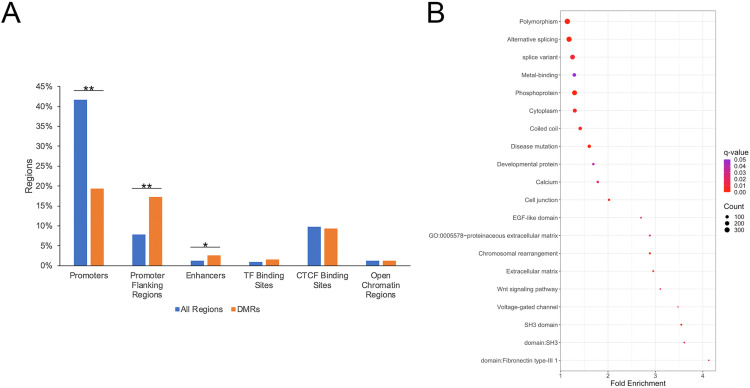
Biological relevance of DMRs. (A) Distribution of all tested regions and DMRs across annotated regulatory regions. (B) Identified biological terms enriched for genes associated with DMRs. * denotes P-value = 0.001. ** denotes P-value < 1 x 10^-22^.

Of the identified DMRs, 369 overlapped with 366 regulatory regions, including 130 promoters, 125 promoter flanking regions, 70 CTCF binding sites, 20 enhancers, 11 transcription factor binding sites, and 10 open chromatin regions (Fig 4A). While significantly fewer DMRs were located in promoter regions than expected by chance (41.63% of tested regions vs. 19.31% of DMRs; P-value = 1.38 x 10^-37^), promoter flanking regions (7.78% of tested regions vs. 17.20% of DMRs; P-value = 4.90 x 10^-23^) and enhancers (1.26% of tested regions vs. 2.60% of DMRs; P-value = 0.001) were significantly enriched for DMRs (Fig 5A). In order to investigate the functional relevance of identified DMRs, differentially methylated regulatory regions located upstream of known genes and DMRs overlapping gene body regions were identified, resulting in the identification of 798 genes associated with DMRs. Functional enrichment analysis indicated differentially methylated genes were associated with Wnt signaling, voltage-gated channels, metal binding, and cellular regulation including alternative splicing, chromosomal rearrangements, and SH3 domain genes (Fig 5B).

### Differential methylation associated with differential expression

Of the DEGs identified, 2 were also differentially methylated in this study (Table 3). *FMN2* displayed increased expression associated with hypermethylation of a DMR located within the gene body in the CCA compared to HCC components. The other differentially methylated DEG was *AC011899.2—*a lncRNA antisense to *PTPRN2*—which also displayed increased expression associated with hypermethylation of a DMR within the gene body in the CCA compared to HCC group. These results suggest the gene body hypermethylation may be related to the increased expression observed for both *FMN2* and *AC011899.2*, consistent with previous studies associating gene body hypermethylation with increased gene expression.

**Table 3:** DEGs associated with DMRs.

Gene	Chr	Start	Stop	Log2 fold change	DMR	DMR location	% methylation difference
AC011899.2	7	157844529	157866092	25.24	7:157864201-157864400	Gene body	26.58
FMN2	1	240004348	240475189	27.85	7:240426801-240427000	Gene body	28.71

Legend: this table displays DEGs associated with DMRs.

### Differentially regulation of canonical Wnt signaling

In this study, 14 genes involved in Wnt signaling were differentially methylated (Table 4), and 1 gene (*FRZB*) displayed increased expression in the CCA compared to HCC components (S2 Table). In total 4 Wnt genes (*WNT3A*, *WNT5A*, *WNT7B*, and *WNT9A*) were differentially methylated in this study. In addition to FZD receptors, LRP5 also functions as a Wnt receptors in the canonical Wnt pathway [38,39], and was hypomethylated in CCA compared to HCC components.

**Table 4:** Differential methylation of Wnt signaling genes.

Gene								DMR (CCA compared to HCC)		Regulatory region			
ID	Chr	Start	Stop	Strand	Chr	Start	Stop	Methylation change	q-value	Chr	Start	Stop	Region
*DISC1*	1	231626815	232041272	+	1	231694001	231694200	27.00%	8.50E-65				
					1	231784001	231784200	31.51%	4.28E-139				
					1	231844001	231844200	28.03%	8.25E-66				
					1	231949201	231949400	32.54%	6.61E-47				
					1	231960401	231960600	26.75%	4.32E-45				
*WNT9A*	1	227918656	227947898	−	1	227928201	227928400	26.22%	3.36E-12				
*WNT3A*	1	228007051	228061260	+	1	228039001	228039200	30.00%	2.71E-15				
*WNT5A*	3	55465715	55490539	−	3	55484001	55484200	−25.54%	7.38E-24				
*CTNND2*	5	10971840	11904043	−	5	11723801	11724000	−32.47%	5.01E-155				
*GRK6*	5	177403204	177442901	+	5	177403401	177403600	−25.11%	8.19E-71	5	177403201	177404000	CTCF Binding Site
										5	177402400	177405001	Promoter
*TLE1*	9	81583683	81689305	−	9	81693201	81693400	−25.87%	6.96E-39	9	81693001	81693400	Promoter Flanking Region
*GRK5*	10	119207589	119459742	+	10	119241001	119241200	28.22%	1.45E-71				
*FRAT1*	10	97319267	97321915	+	10	97308601	97308800	25.79%	4.71E-27	10	97307201	97309399	Promoter Flanking Region
*LRP5*	11	68312609	68449275	+	11	68381601	68381800	−31.21%	2.62E-15				
*TLE3*	15	70047790	70098176	−	15	70062401	70062600	−27.33%	2.50E-289				
*APCDD1*	18	10454628	10489948	+	18	10485201	10485400	27.18%	3.63E-33				
*TLE5*	19	3052910	3063107	−	19	3055601	3055800	−42.29%	3.29E-139				
					19	3055801	3056000	−35.41%	1.32E-266				
*WNT7B*	22	45920362	45977129	−	22	45932401	45932600	26.52%	4.61E-32				

Legend: this table displays differential methylation of Wnt signaling genes.

Differential methylation of several regulators of Wnt signaling was also observed, including hypermethylation of a promoter flanking region located upstream of *FRAT1* (Table 4). Hypomethylation of the promoter region and CTCF binding site overlapping the *GRK6* TSS was observed in CCA compared to HCC samples, in addition to hypermethylation of *GRK5* in the CCA compared to HCC samples. *CTNND2* was also hypomethylated in CCA compared to HCC samples. In addition, 5 DMRs located within *DISC1* were hypermethylated in the CCA compared to HCC samples. Altered methylation and expression of negative regulators of Wnt signaling was also identified, including increased expression of *FRZB* in CCA compared to HCC components (Log2 fold change 5.6, q-value = 0.043). In addition, hypomethylation of several TLE genes (*TLE1, TLE3,* and *TLE5*) was observed in CCA compared to HCC samples, including hypomethylation of a region located within the *TLE1* promoter flanking region (Table 4).

### Differentially regulation of non-canonical Wnt signaling

*WNT5A*, which initiates the Wnt/PCP and the Wnt/Ca^2+^ pathways, was hypermethylated in the HCC compared to CCA samples (Table 4). In this study, altered expression of genes involved in Ca^2+^ and other ion transport was observed, including increased expression of *ATP2C2* in CCA compared to HCC components (S3 Table). Increased expression of the K-Cl cotransporter *SLC12A6* was also observed in the CCA compared to HCC samples. Increased expression of three olfactory receptors (*OR10J1*, *OR6N2*, and OR4F17) was also observed in CCA compared to HCC samples. Together, differential methylation of *WNT5A*, in addition to differential methylation and expression of genes associated with voltage-gated channels, calcium, and metal binding were identified (Fig 5B; S3 Table).

## Discussion

This study presents a comprehensive analysis of transcriptional and DNA methylation profiles in combined HCC-CCA tumors micro-dissected from FFPE samples. Despite the known challenges associated with the use of FFPE tissue samples, analysis of RNA sequencing data identified 57 differentially expressed genes while RRBS data revealed 808 DNA regions that were differentially methylated in the CCA component as compared to the HCC component. Increased expression of genes associated with tumor progression and aggressiveness as well as genes associated with poor treatment response were identified in the CCA tissue, providing a biological basis for the poor prognosis observed clinically. Differential methylation in promoter flanking and enhancing regions were also noteworthy, including genes that associated with Wnt signaling, voltage-gated channels, metal binding, and cellular regulation. In addition, gene body hypermethylation associated with increased *FMN2* and *AC011899.2* expression was observed consistent with previous studies associating gene body hypermethylation with increased gene expression. Both genes are associated with increased tumor progression through various mechanisms.

With respect to differential expression associated with individual HCC-CCA components, several genes identified in this study have been shown to influence cancer outcomes, and warrant consideration. *LPAR3* is associated with HCC progression, prognosis, and invasiveness [40]. *NEFL*, upregulated in CCA compared to HCC components, was previously identified as a potential prognostic marker for CCA recurrence-free survival [41]. *TUSC7* expression suppresses epithelial-to-mesenchymal transitions in HCC, with reduced expression reported to be a predictor of poor survival [42]. *NANOG* expression is associated with poor overall survival and disease-free survival in HCC and CCA [43,44]. *TYRO3* is associated with HCC tumor progression and sorafenib resistance, with increased sorafenib sensitivity observed following *TYRO3* knockdown *in vitro* [45,46]. *BRWD1* is involved in chromatin remodeling, is activated in *TP53* mutated HCC [47], and promotes cisplatin resistance in cervical cancer [48]. Upregulation of *TTBK2* is observed in kidney carcinoma and melanoma and is associated with resistance to the tyrosine kinase inhibitor sunitinib [49].

As it pertains to differential methylation, several DMRs identified in this investigation warrant attention. *FMN2* facilitates movement of chromatin and repair factors after DNA damage [50], protects cells from apoptosis, and plays a role in stress-induced cell cycle arrest [51]. Increased *FMN2* expression has also been shown to promote cancer cell migration and invasion [52]. *PTPRN2* is significantly overexpressed in a subset of tumors, including colon, prostate, pancreas, and breast cancers [53]. In addition, *AC011899.2* expression has been shown to be predictive of patient prognosis for low-grade gliomas [54].

Increased expression of all 4 Wnt genes has been observed in HCC and CCA samples leading to activation of Wnt signaling [55–58]. Binding of Wnt proteins to FZD receptors results in translocation of CTNNB1 to the nucleus where it triggers activation of multiple intracellular signaling cascades [55]. *FRAT1* is a positive regulator of Wnt signaling, and represents a potential HCC therapeutic target due to its ability to promote hypoxia-induced HCC cancer progression and metastasis [59]. Both *GRK6* and *GRK5* have been shown to regulate canonical Wnt signaling [60], with both genes implicated in increased cellular proliferation, migration, and aggressiveness in a variety of cancer types [61]. *CTNND2* regulates canonical Wnt signaling and functions as an oncogene by promoting HCC cell proliferation and migration [62–64]. *DISC1* promotes Wnt-mediated neural progenitor proliferation by acting as a positive regulator of CTNNB1 abundance [65]. While the role of *DISC1* in tumorigenesis has not been well studied to date, studies have demonstrated its ability to promote non-small cell lung cancer cell proliferation while inhibiting glioblastoma cell proliferation [66,67]. *FRZB* expression is observed in a subset of HCC tumors, with elevated expression associated with HCC bone metastasis [68]. The TLE gene family of transcriptional co‐repressors has been identified as repressors of Wnt target gene expression [69].

In addition to canonical Wnt signaling, a number of studies have demonstrated activation of non-canonical Wnt signaling in HCC [70,71], specifically through the Wnt/PCP and the Wnt/Ca^2+^ pathways [72,73]. Activation of this pathway through WNT5A results in increased Ca^2+^ influx and intracellular levels leading to inhibition of canonical Wnt signaling.

*ATP2C2* results in increase Ca^2+^ influx and promotion of cancer cell motility and proliferation [74,75]. Upregulation of *SLC12A6* has been observed in several cancer types and is associated with tumor progression and poor prognosis [76,77]. Finally, although olfactory receptors were initially discovered due to their role in smell, increased olfactory receptor expression is observed across several cancer types associated with Ca^2+^ signaling and reduced cellular proliferation [78].

Increasing evidence has shown that the Wnt signaling pathway plays a vital role in HCC [55], especially the canonical Wnt pathway [79]. Wnt signaling plays a crucial role in the regulation of diverse processes, including cell proliferation, survival, migration, and embryonic development [80]. In addition, aberrant Wnt signaling has been implicated in development and progression of numerous cancer types, including HCC [81] and CCA [56]. Approximately 95% of HCC cases display altered Wnt signaling [71], with abnormal regulation of the canonical Wnt signaling pathway considered a major driver of HCC development [82]. In addition, recent studies have also demonstrated the importance of Wnt signaling in CCA progression, with inhibition of the canonical Wnt pathway resulting in reduced tumor burden [56]. Significant overexpression of LPAR3 has been identified in HCC tumor margins and was interestingly overexpressed in the CCA portion of combined HCC-CCA, potentially owing to the increased aggressiveness along with *NEFL* and *TUSC7* that were also overexpressed in the CCA portions [83].

The altered expression of genes implicated in resistance to chemotherapeutics employed to treat HCC and HCC-CCA clinically could plausibly explain differences in therapeutic outcomes between HCC and HCC-CCA patients, highlighting the importance of accounting for underlying HCC and CCA tumor biology when determining the optimal course of treatment in clinical practice, while preventing ineffective treatment with resistant drugs. To this end, several genes associated with drug resistance were also overexpressed in the CCA portion of tumors, including *TYRO3*, *BRWD1*, *TTBK2*, which are implicated in sorafenib, cisplatin, and sunitinib resistance, respectively. Differential methylation of *WNT5A*, in addition to differential methylation and expression of genes associated with voltage-gated channels, calcium, and metal binding suggests differential activation of both canonical Wnt and Wnt/Ca^2+^ signaling between HCC and CCA components. As Wnt inhibitors and other approaches for targeting of the Wnt signaling pathway show promise for cancer treatment, improved understanding of the differential role of Wnt signaling in HCC and CCA progression could lead to more effective combination therapies for this deadly disease.

This study has limitations. First, the investigation had a retrospective, single center design with a small cohort of patients with HCC-CCA; owing to the rarity of this disease, accrual of large sample sizes for prospective evaluation is a clinical challenge. Second, the of FFPE specimens resulted in high levels of RNA degradation in tissue samples. Coupled with the inability to distinguish HCC and CCA components based on DEGs, further studies are required to confirm gene expression differences identified in this study. Third, this investigation did not include an assessment of therapeutic strategies employed and did not make any associations between tumor genomic profile and treatment outcomes, which would be considerably limited by the small patient sample studied.

In conclusion, the increased expression of several genes associated with tumor progression and aggressiveness in CCA samples suggests a more aggressive nature of CCA compared to HCC. In all, these results provide insights into the epigenetic regulatory patterns associated with the two components of combined HCC-CCA. Future studies may aim to understand the effects of epigenetic regulation on treatment response for this deadly disease.

## Supporting information

S1 Table RNA-seq alignment and gene expression. This table displays RNA-seq alignment and gene expression.
(XLSX)

S2 Table RRBS alignment rates. This table displays RRBS alignment rates.
(XLSX)

S3 Table DEGs identified between HCC and CCA components. This table displays DEGs identified between HCC and CCA components.
(XLSX)

S4 Table DMRs identified between HCC and CCA components. This table displays DMRs identified between HCC and CCA components.
(XLSX)
